# Trends in tricyclic antidepressant prescribing and poisoning in England and Wales 2016–2020

**DOI:** 10.1111/bcp.16400

**Published:** 2025-01-29

**Authors:** Laurence Gray, Michael Beddard, Stephen Jones, Asiyah Begum, Noraini B. Azhar, Paul Deslandes, James Coulson, Sally Bradberry, Euan A. Sandilands, Ruben H. Thanacoody, Matthew O. Ivory

**Affiliations:** ^1^ National Poisons Information Service Cardiff University Hospital Llandough Cardiff UK; ^2^ Centre for Healthcare Evaluation, Device Assessment and Research (CEDAR) Cardiff and Vale University Health Board Cardiff UK; ^3^ School of Pharmacy and Pharmaceutical Sciences Cardiff University Cardiff UK; ^4^ All Wales Therapeutics and Toxicology Centre, NHS Wales Cardiff UK; ^5^ Faculty of Life Sciences and Education Department University of South Wales Pontypridd UK; ^6^ Clinical Pharmacology Cardiff University Cardiff UK; ^7^ NPIS Birmingham, National Poisons Information Service Birmingham UK; ^8^ NPIS Edinburgh, National Poisons Information Service Edinburgh UK; ^9^ NPIS Newcastle, National Poisons Information Service Newcastle UK

**Keywords:** antidepressants, England, poisoning, prescribing, tricyclic, Wales

## Abstract

**Aims:**

Tricyclic antidepressants (TCAs) are commonly prescribed despite no longer being a NICE‐recommended first‐line treatment for depression and their recognized toxicity in overdose. This study examined prescribing, mortality, hospital admissions and clinical TCA data to quantify the use and impact of TCAs in England and Wales.

**Methods:**

Primary care prescription data for the eight TCAs currently licensed in England and Wales were analysed alongside hospital admission and mortality data relating to TCAs over the study period (January 2016–December 2020 inclusive). Monthly Toxbase™ accesses regarding TCAs during the study period for each TCA were quantified. National Poisons Information Service (NPIS) enquiry data involving TCA exposure were obtained and patient demographics, circumstance, dose ingested and poisoning severity were analysed.

**Results:**

English and Welsh mean monthly TCA prescriptions per 100 000 people significantly increased during the study period, both driven by amitriptyline 10 mg tablets (95% confidence interval [CI] 3.49–4.59 and 6.36–7.92, respectively). Deaths from poisoning where a TCA was mentioned on the death certificate fell. Toxbase™ accesses increased for amitriptyline and nortriptyline but decreased for all other TCAs. NPIS telephone enquiries relating to TCAs decreased. Hospital admission data did not demonstrate an increase in admissions related to TCAs.

**Conclusions:**

Reduced TCA poisoning mortality in England and Wales was seen despite increased dispensing of TCAs in both nations. The prescribing of low‐dose amitriptyline formulations was associated with increased consultation with Toxbase™ but not increased hospital admissions or NPIS enquiries, suggesting a fall in TCA poisoning severity resulting from their changing pattern of usage.

**What is already known about this subject**

- Tricyclic antidepressants (TCAs) are no longer first‐line therapy in the treatment of depression in the United Kingdom (UK) due to their toxicity in overdose but remain widely used for other indications including neuropathic pain.
- Within the UK, England and Wales have similar but differing healthcare structures and have used different interventions to try to reduce TCA harm.
- There has not been a comprehensive review of TCA usage and impacts across England and Wales following these interventions.

**What this study adds**

- Primary care TCA dispensing increased over the study period, driven by low strength amitriptyline formulations, with nortriptyline the only other TCA to show an increase in overall dispensing 2016–2020.
- National Poisoning Information Service (NPIS) poisoning enquiries, hospital admissions, and Office for National Statistics (ONS)‐recorded deaths related to TCAs all decreased in both England and Wales over the study period.
- Wales had greater TCA dispensing but fewer poisonings and deaths per 100 000 people than England over the data period.

## 1 INTRODUCTION

Tricyclic antidepressant (TCA) utilization in England and Wales has changed over the last two decades.^1^, ^2^ National Institute of Health and Care Excellence (NICE) Guidelines published in 2004, 2009 and 2022 in the UK have all recommended selective serotonin reuptake inhibitors (SSRIs) and serotonin‐norepinephrine reuptake inhibitors (SNRIs) as the first‐line pharmacological treatment for depression instead of TCAs.^3^, ^4^, ^5^ These newer agents possess improved tolerability and safety profiles compared to TCAs.^6^, ^7^ Despite this, in the UK, TCAs are still used in the treatment of depression in patients already on these medications or where other pharmacological treatments have been unsuccessful.^3^, ^4^, ^5^ Amongst the other indications for TCAs (Table 1), amitriptyline is licensed for the treatment of neuropathic pain^8^ and is used off‐licence in chronic pain,^9^ whilst nortriptyline is used for neuropathic pain but is not licensed for this indication.^10^ The doses recommended in the British National Formulary (BNF) for depression are higher than those for neuropathic pain for both amitriptyline and nortriptyline.^10^ Chronic pain is the second most common indication for TCAs after depression,^11^ though a recent Cochrane systematic review found no high‐quality or high‐certainty evidence for the efficacy of amitriptyline for this indication.^12^

**TABLE 1:** Licensed and unlicensed indications of tricyclic antidepressants as stated in the British National Formulary, British National Formulary for Children and Summaries of Product Characteristics (SmPC) documentation.

Tricyclic antidepressant	UK licensed indication(s)	UK unlicensed indication(s)
Amitriptyline	Major depressive disorder^a^ Neuropathic pain^a^ Migraine prophylaxis^a^ Chronic tension‐type headache prophylaxis^a^ Nocturnal enuresis^b^	Abdominal pain or discomfort in patients who have not responded to laxatives, loperamide, or antispasmodics^a^ Emotional lability in multiple sclerosis^a^ Neuropathic pain (in children)^c^ Chronic pain^d^
Clomipramine	Depressive illness^a^ Phobic and obsessional states^a^ Adjunctive treatment of cataplexy associated with narcolepsy^a^	N/A
Dosulepin (Dothiepin)	Depressive illness^a^	N/A
Doxepin	Depressive illness^a^ Pruritus in eczema^a^	N/A
Imipramine	Depressive illness^a^ Nocturnal enuresis^a^	Attention deficit hyperactivity disorder^a^
Lofepramine	Depressive illness^a^	N/A
Nortriptyline	Depressive illness^a^ Nocturnal enuresis^e^	Neuropathic pain^a^
Trimipramine	Depressive illness^a^	N/A

^a^ Joint Formulary Committee. BNF. 82nd ed. London: BMJ Group; Pharmaceutical Press, 2021.

^b^ Brown & Burk UK Ltd. Summary of Product Characteristics (SmPC): Amitriptyline 10 mg Film‐Coated Tablets. Electronic Medicines Compendium (EMC). Published November 4, 2023. Accessed September 26, 2024. https://www.medicines.org.uk/emc/product/10849/smpc.

^c^ Paediatric Formulary Committee. BNF for Children. 2020–2021 Edition. London: BMJ Group; Pharmaceutical Press, 2020.

^d^ NICE. NG193: Chronic pain (primary and secondary) in over 16s: assessment of all chronic pain and management of chronic primary pain. London: National Institute for Health and Care Excellence (NICE), 2021.

^e^ Flamingo Pharma (UK) Ltd. Summary of Product Characteristics (SmPC): Nortriptyline 10 mg Tablets. Electronic Medicines Compendium (EMC). Published: April 8, 2022. Accessed: September 26, 2024. https://www.medicines.org.uk/emc/product/13516/smpc.

The narrow therapeutic index of TCAs creates a risk of accidental overdose in patients.^13^ This risk exists alongside the higher incidence of intentional overdoses amongst patients with depression.^14^, ^15^ Tricyclic and tetracyclic antidepressants were amongst the ten most common poisoning substances from 1998 to 2014 in 10‐ to 24‐year‐olds across England.^16^ A higher ratio of deaths to prescriptions for TCAs than other classes of antidepressants has also been reported.^6^, ^17^ The safety of TCAs in general and dosulepin in particular has been questioned and is the subject of drug safety updates by the Medicines and Healthcare Product Regulatory Agency (MHRA)^18^ and also the introduction of a prescribing indicator in Wales.^19^, ^20^

Toxicity in TCA overdose is due to a combination of altered reuptake of pre‐synaptic noradrenaline and serotonin coupled with antagonism of histaminergic, muscarinic and α‐1 adrenergic receptors.^21^ This leads to the altered mental status, neuromuscular hyperactivity, autonomic instability and anticholinergic features such as dry mouth, delirium and urinary retention seen in TCA overdose. Potent sodium channel‐blocking properties can lead to life‐threatening arrhythmias and seizures.^22^ Extended hospital stays and prolonged observation may be required as TCAs can have peak plasma concentrations at 8 h post‐ingestion, and an elimination half‐life of up to 39 h.^23^, ^24^

Primary care prescribing and associated deaths data for TCAs in England and Wales have been reported previously. Between 1993 and 1997 there were approximately 53 deaths per million TCA prescriptions, with TCAs involved in over 95% of all antidepressant deaths reported in England and Wales.^25^ In the period 1998–2000, English and Welsh TCA‐related deaths fell but still made up over 85% of the antidepressant deaths observed.^26^ More recent studies have focused solely on English data.^27^, ^28^, ^29^, ^30^ This study provides comprehensive insight into primary care dispensing of TCAs in both England and Wales over the five‐year period 2016–2020 alongside unintended outcomes of this prescribing, beyond that of previous reports.^6^, ^31^, ^32^ Cases of poisoning, hospital admissions and mortality across the two UK nations were quantified and the relationship with prescribing patterns considered. We integrated clinical data from UK National Poisons Information Service (NPIS) TCA poisoning enquiries alongside Toxbase™ accesses by clinicians to provide insight into trends in TCA poisoning case numbers, their severity and clinical management. It is intended that this study will provide baseline data for the years up to and including the onset of the COVID‐19 pandemic that will contextualize ongoing work elsewhere into mental health impacts and inform clinical guidelines.

## 2 METHODS

### 2.1 Data sources

Figure S1 summarizes the datasets utilized within this study.

#### 2.1.1 Population data

Mid‐year population estimates for England and Wales were obtained from the Office of National Statistics (ONS) ‘*Population estimates for the UK, England and Wales, Scotland and Northern Ireland*’ publications for the years 2015–2020. Population figures for individual months and financial years were calculated using weighted means of the mid‐year population estimates where necessary to align with the timescales of data from other sources.

#### 2.1.2 Primary care prescription data

Primary care prescription data (January 2016–December 2020 inclusive) were collected for the eight TCA drugs currently licensed in the UK: amitriptyline, clomipramine, dosulepin (dothiepin), doxepin, imipramine, lofepramine, nortriptyline and trimipramine. English data were obtained from the Electronic Prescribing Analysis and Cost Tool (ePACT), whilst Welsh data were obtained from the Comparative Analysis System for Prescribing Audit (CASPA). Each dataset represents all NHS prescriptions that were dispensed and sent for reimbursement by a pharmacy in each nation each month. The measure used in the study was ‘items’, equivalent to the number of instances a formulation containing each drug had been dispensed. To focus analysis solely on oral single‐agent TCA formulations, the study excluded topical and combination TCA preparations which accounted for 191 items or 0.0002% of the items dispensed over the study period.

#### 2.1.3 Secondary care data

Hospital admission data from the Hospital Episode Statistics (HES) and the Patient Episode Database for Wales (PEDW) were analysed for England and Wales, respectively. In each dataset, finished consultant episodes—‘the time a patient spends in the continuous care of one consultant using hospital site or care home bed(s) of one health care provider’^33^—under the primary diagnosis code T430 (Poisoning: Tricyclic and tetracyclic antidepressants) were used as the measure of TCA‐related hospital episodes. The T430 code is derived from the International Classification of Diseases (ICD‐10) system, which is adopted in the HES/PEDW datasets to provide a consistent and standardized approach to recording diagnoses in hospital admissions.

#### 2.1.4 Mortality data

Mortality data comprising both single‐agent and multi‐agent poisoning deaths from 2016 to 2020, where an antidepressant, any TCA or specifically amitriptyline or dothiepin (dosulepin) was mentioned on the death certificate were extracted from the relevant ONS dataset.^34^ This dataset contains deaths coded according to the ICD‐10 system. Additionally, data on TCA involvement in suicide/non‐suicide deaths were obtained from a bespoke dataset commissioned from the ONS, which also utilizes coded data for analysis.^35^

#### 2.1.5 Toxbase™ accesses

The NPIS manage Toxbase™, a database providing clinical toxicology advice to healthcare professionals that is accessible via website or app. The number of monthly website and app visitor accesses in England and Wales between January 2016 and December 2020 for each UK‐licensed TCA was provided by the NPIS.

#### 2.1.6 United Kingdom poisons information database (UKPID)

The UKPID database contains clinical details of all telephone enquiries to the NPIS, including demographic data, poisoning circumstance, substance data, clinical features, treatments and outcomes. This dataset uses set categories for each variable with a separate field for free text input. UKPID enquiry data for the period January 2016–December 2020 inclusive were analysed. Enquiries regarding adverse drug reactions, ethanol co‐ingestion, calls from a member of the public and general information enquiries were excluded. Enquiries relating to cases in Scotland and Northern Ireland were also excluded. Duplicate case enquiries were identified and merged so that each case represented a unique poisoning event.

##### Ingested dose

The ingested dose (as milligram of drug per kilogram of patient bodyweight) was calculated for each single‐agent case. Of the 775 single‐agent TCA poisoning cases, there were a total of 272 instances where bodyweight information was not documented. In such cases, the weight of 58 kg for adult females, 68 kg for adult males or appropriate weight for children based on their sex and age was used, as per British National Formulary (BNF) guidance.^10^ Of the 775 cases, the ingested dose was known in 678 (87.5%) cases. Cases where the ingested dose was not known were excluded from the analysis.

##### Poisoning severity score

Poisoning severity score (PSS)^36^ is recorded to grade the severity of the poisoning for each case. PSS scores and symptom severity are categorized as follows: 0 = none; 1 = minor; 2 = moderate; 3 = severe; 4 = fatal. A ‘MaxPSS’ score was allocated to represent the highest grade of poisoning severity experienced by a patient during a poisoning event.

### 2.2 Statistical tests

Statistical tests, unless otherwise indicated, were carried out using SPSS Statistics for Windows, Version 27.0 (IBM Corp., Armonk, NY). Ingested dose data were analysed using a one‐way analysis of variance (ANOVA) with Tukey's post hoc analysis. Time series analyses were performed on primary care prescribing data and Toxbase™ accesses data using R Statistical Software v4.2.1 (R Core Team, Vienna, Austria, 2022) with tsibble,^37^ dplyr^38^ and forecast^39^ packages. Plots were produced using the package ggplot2.^40^ To visualize trends in monthly primary care dispensing data for individual TCAs and compare dispensing trends in England and Wales, seasonal and trend decomposition using LOESS (STL) was performed.^41^ STL decomposes time series data into trend, seasonal and random components. Trend plots illustrate presence (or absence) of trend, its direction (increasing or decreasing) and provides a measure of trend strength on a scale of 0 (no trend) to 1 (strongest possible trend). To explore trends further, ordinary least squares regression was performed using the tslm function of the forecast package in R. This function fits linear models to time series, including trend and seasonal components. As there is some evidence for seasonality in the data series, models were developed which effectively included seasonal (monthly) dummy variables. Coefficients of regression for trend (with 95% CI) were obtained which describe the monthly increase or decrease. Primary care dispensing data were analysed as items per 100 000 population for England and Wales.

### 2.3 Ethics statement

Ethical approval was not required as this study used publicly available data, statistical data and anonymized information which was routinely collected during episodes of clinical care (http://www.hra-decisiontools.org.uk/ethics/).

### 2.4 Nomenclature of targets and ligands

Key protein targets and ligands in this article are hyperlinked to corresponding entries in http://www.guidetopharmacology.org, and are permanently archived in the Concise Guide to PHARMACOLOGY 2023/24.^42^

## 3 RESULTS

### 3.1 Primary care dispensing of TCAs

**FIGURE 1:**
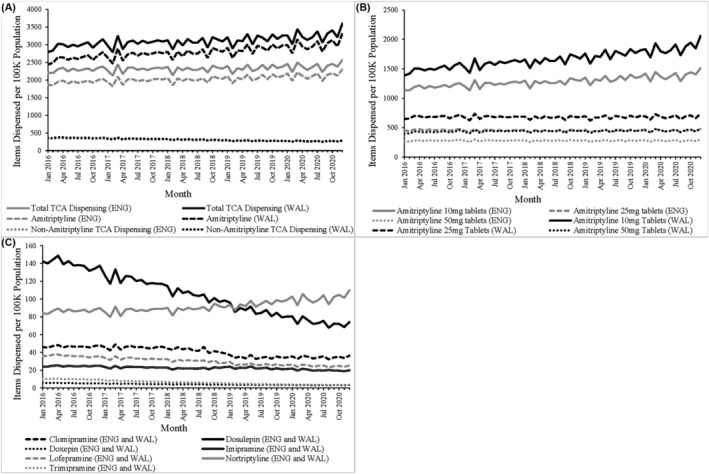
Primary care tricyclic antidepressant (TCA) dispensing in England (ENG) and Wales (WAL) 2016–2020. (A) Total TCA items dispensed each month in primary care, alongside a breakdown of amitriptyline and non‐amitriptyline items scaled to 100 000 population within each nation. (B) Population‐scaled monthly English and Welsh amitriptyline items dispensed classified by tablet strength. (C) Combined English and Welsh non‐amitriptyline TCA items dispensed, scaled to the combined populations of the two nations.

The primary care dispensing of TCA items per 100 000 population was higher and increased more quickly in Wales compared to England between 2016 and 2020, largely driven by increased dispensing of amitriptyline (Figure 1). Amitriptyline accounted for 90.0% and 87.1% of TCA items dispensed in Wales and England respectively, with a mean monthly total of 1.22 million items across the two nations. Dispensing of amitriptyline 10 mg tablets per 100 000 population increased by 4.04 items per month (95% CI 3.49–4.59, *P* < 0.001) in England and 7.31 items per month (95% CI 6.36–7.92, *P* < 0.001) in Wales (Figure 1; Figure S2). There was no significant trend in the dispensing of amitriptyline 25 mg tablets in Wales (*P* = 0.08) and a decrease by 0.2 items per month in England (95% CI 0.39– −0.01, *P* = 0.043). Dispensing of 50 mg tablets also showed weak trend strengths (0.37 for Wales, 0.03 for England) though there was a significant positive linear trend in Wales with items increasing by 0.4 items per month per 100 000 population (95% CI 0.25–0.65, *P* < 0.001) (Figure S2).

Trends of increasing numbers of items dispensed per 100 000 population were observed for amitriptyline and nortriptyline only, with all other TCAs showing a trend of decreasing dispensing over the data period (Figure 1; Figure S3 and Table S1). Nortriptyline dispensing increased by 0.07 (95% CI 0.03–0.11, *P* < 0.001) and 0.32 (95% CI 0.27–0.37, *P* < 0.001) items per 100 000 population per month in Wales and England respectively, with the greatest increases in both nations occurring post‐2018.

Following scaling to population size, dispensed prescriptions of clomipramine, doxepin and lofepramine in Wales exceeded those in England throughout the study period with the rates of decrease in dispensing being similar in both nations for each drug. Dispensing rates of imipramine in England exceeded those in Wales throughout the study period. In January 2016, dispensing of dosulepin in England exceeded Wales but the rate of decrease in England of 1.4 per month (95% CI −1.37– −1.47, *P* < 0.001) was significantly greater than the decrease in Wales of 0.93 per month (95% CI −0.88– −0.98, *P* < 0.001). Consequently, by the end of the study period, population‐scaled dosulepin items were lower in England than Wales.

### 3.2 ONS cause of death data

**FIGURE 2:**
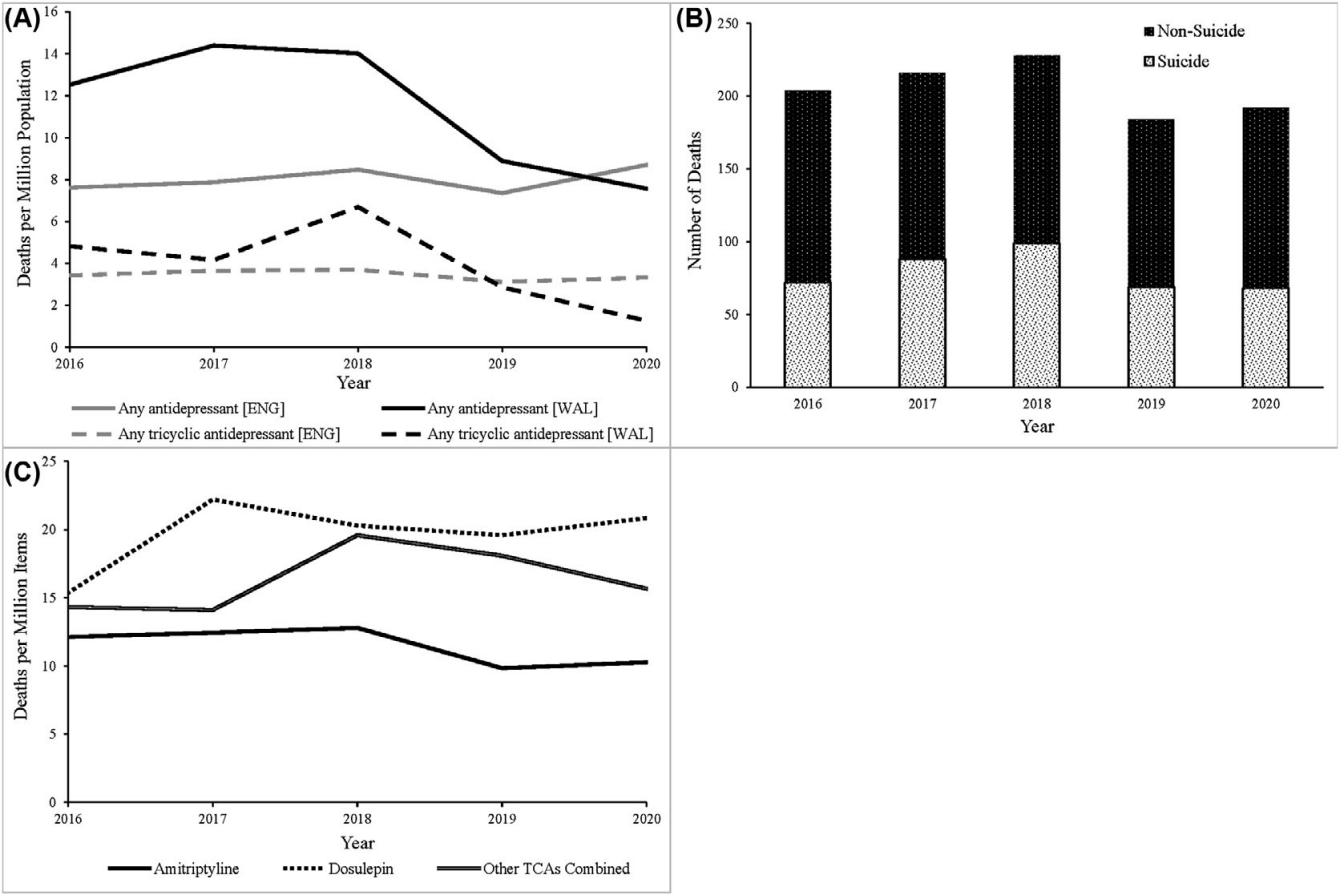
Deaths data for England and Wales reported by ONS 2016–2020. (A) Deaths per million population in England and Wales recorded as involving an antidepressant drug and the proportion of these deaths where this drug was a tricyclic antidepressant (TCA). (B) Proportion of annual TCA poisoning deaths (England and Wales combined) ascribed as suicide and non‐suicide. (C) Deaths recorded as involving a TCA each year (England and Wales combined) per million items dispensed of the corresponding TCA(s) across the two nations in that year.

Whilst overall deaths per million population where any antidepressant was mentioned on the death certificate were initially higher in Wales (12.5) than England (7.6), a sharp decrease in antidepressant poisoning deaths in Wales from 2018 onwards meant that these deaths were higher per million population in England at the end of the data period (Figure 2). This change represented a 14.2% increase in England and a 39.6% decrease in Wales in 2020 compared to 2016 (an increase in total deaths by 71 and a fall of 15 respectively).

Where a TCA was a contributory factor in deaths, Wales had more deaths per million population in 2016 but by 2020 this had fallen below the rate in England. Both nations had fewer TCA‐related deaths in 2020 than 2016, but the magnitude of the change was greater in Wales (*n* = 11; a 73.9% decrease in population‐scaled deaths) than England (*n* = 1; a 2.9% decrease in population‐scaled deaths).

In the UK, cause of death classifications include both intentional self‐harm and events of undetermined intent as suicides, while non‐suicide deaths encompass accidental poisonings, adverse drug reactions and natural causes where the medication may have played a role.^43^ Amongst deaths where a TCA was mentioned on the death certificate, there were more non‐suicide deaths (*n* = 628) than suicide deaths (*n* = 396) (Figure 2), with the proportion of TCA deaths ascribed as suicides varying from 35.3% to 43.4% across the 5 years. Female suicide deaths exceeded male suicide deaths across the data period (*n* = 212 and *n* = 184 respectively), whilst non‐suicide deaths were evenly split by sex (both *n* = 314). Whilst amitriptyline was cited in 82.0% of TCA deaths 2016–2020, there were fewer deaths per million items dispensed than those involving dosulepin or the remaining six UK‐licensed TCAs (Figure 2). Amongst the three TCA categories recorded by the ONS, only amitriptyline showed a decrease in deaths per million items between 2016 and 2020. Dosulepin showed the highest mortality relative to items dispensed across all years of the data period as well as the greatest net increase, increasing from 15.4 deaths per million items dispensed in 2016 to 20.8 in 2020.

### 3.3 NPIS enquiries

#### 3.3.1 TCA agent enquiries

**FIGURE 3:**
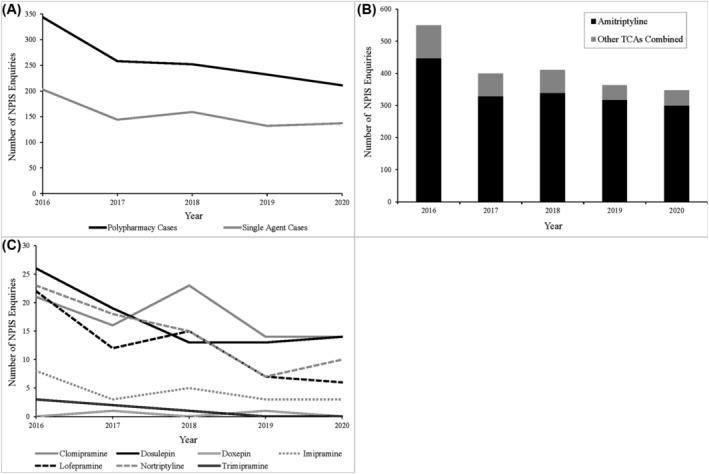
Agents involved in National Poisons Information Service (NPIS) tricyclic antidepressant (TCA) phone enquiries 2016–2020. (A) The annual number of NPIS phone enquiries that involved single TCA agent poisonings *vs.* those where multiple agents (at least one TCA in combination with other TCA and/or non‐TCA agent(s)) were involved. (B) The number of times that TCA agents were recorded in NPIS poisoning phone enquiries annually between 2016 and 2020 (single‐agent and polypharmacy combined) broken down into amitriptyline and the remaining seven UK‐licensed TCAs combined. (C) Breakdown of the annual occurrences of each non‐amitriptyline TCA's involvement in NPIS phone enquiries.

Between 2016 and 2020, there were a total of 2069 enquiries to the NPIS where a TCA was reported. Amongst these, 62.5% (*n* = 1294) were polypharmacy enquiries and the remaining 37.5% (*n* = 775) were single‐agent poisonings (Figure 3). Total annual enquiries decreased over the data period, with polypharmacy and single‐agent poisonings falling by 38.7% and 32.5% respectively in 2020 compared to 2016.

The number of mentions of TCAs in the UKPID dataset between 2016 and 2020 decreased from 550 in 2016 to 347 in 2020 (Figure 3). All eight TCAs licensed in the UK were involved in at least one case recorded in the UKPID during this period. Amitriptyline was the most frequently reported, mentioned in 83.6% (*n* = 1733) of all cases. The next most frequently recorded TCAs were clomipramine and dosulepin (4.2% and 4.1% of TCA mentions respectively) (Figure 3). The least commonly identified TCAs were trimipramine and doxepin (0.3% and 0.1% of all TCA mentions respectively).

**FIGURE 4:**
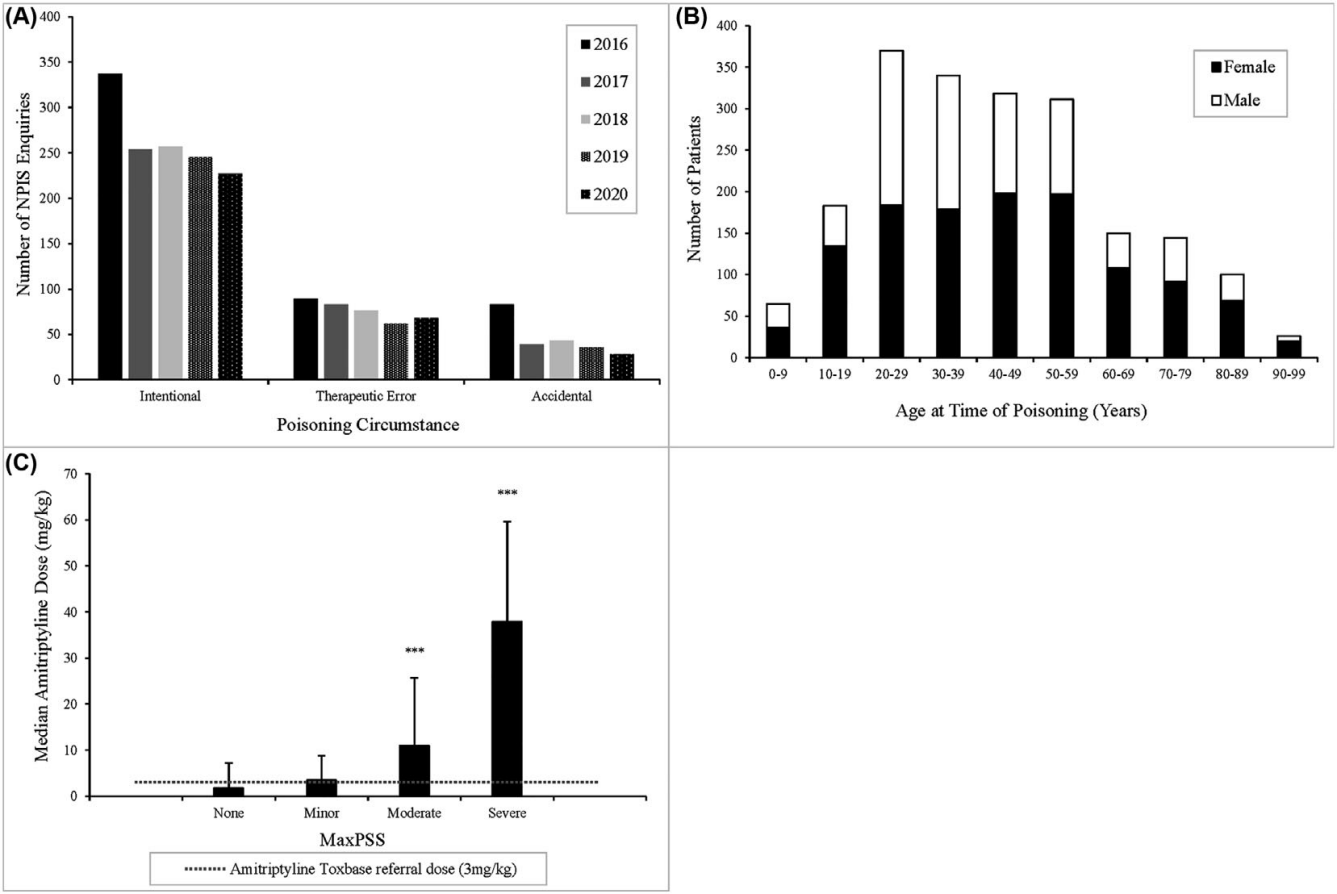
Demographic and clinical analysis of National Poisons Information Service (NPIS) tricyclic antidepressant (TCA) phone enquiries 2016–2020. (A) The three most frequent poisoning circumstances recorded. (B) Sex/age of patients involved in TCA poisoning cases. (C) Analysis of amitriptyline dose ingested (mg kg^−1^) *vs.* MaxPSS recorded for poisonings with the Toxbase™ amitriptyline referral dose indicated (*** = *P* < 0.001).

Intentional poisonings were the most common circumstance seen in the TCA‐related NPIS phone enquiries, with therapeutic error and accidental poisonings representing the second and third most common circumstance respectively (Figure 4). The number of intentional poisonings exceeded the number of poisonings due to all other circumstances combined in all years of the data period. In cases where sex was recorded (*n* = 2039), a higher proportion of female patients (58.8%) than male patients were seen (Figure 4). Poisoning incidents increased with the age of patients up to a peak in 20–29‐year‐olds, which was the only age bracket in which male patients outnumbered female patients. A steady fall was then seen up to 60–69 years, where poisoning incidents fell by 51.8% compared to the preceding age bracket. Four deaths were recorded, involving three females and one male. Ages were available for three cases with a mean patient age of 29 years. The ingested agents were amitriptyline (*n* = 3) and nortriptyline (*n* = 1), with doses documented in two cases: 1.25 g of amitriptyline and 6 g of nortriptyline. Cardiac arrest was reported in all cases.

#### 3.3.2 Maximum poisoning severity score (MaxPSS)

To exclude contributory effects of co‐ingested agents in poisonings, including ethanol, single‐agent TCA poisonings were analysed for the MaxPSS the patient experienced. The MaxPSS was known at the time of the enquiry and recorded in the UKPID database for 729 (94%) of these cases. The median ingested dose for amitriptyline cases with a MaxPSS score of ‘none’ (1.75 mg kg^−1^) was below the Toxbase™ referral dose of 3 mg kg^−1^ while the median dose exceeded the referral threshold of 3 mg kg^−1^ for all symptomatic scores: minor (3.45 mg kg^−1^), moderate (11.01 mg kg^−1^) and severe (37.93 mg kg^−1^) (Figure 4).

### 3.4 Toxbase™ website/app access data

**FIGURE 5:**
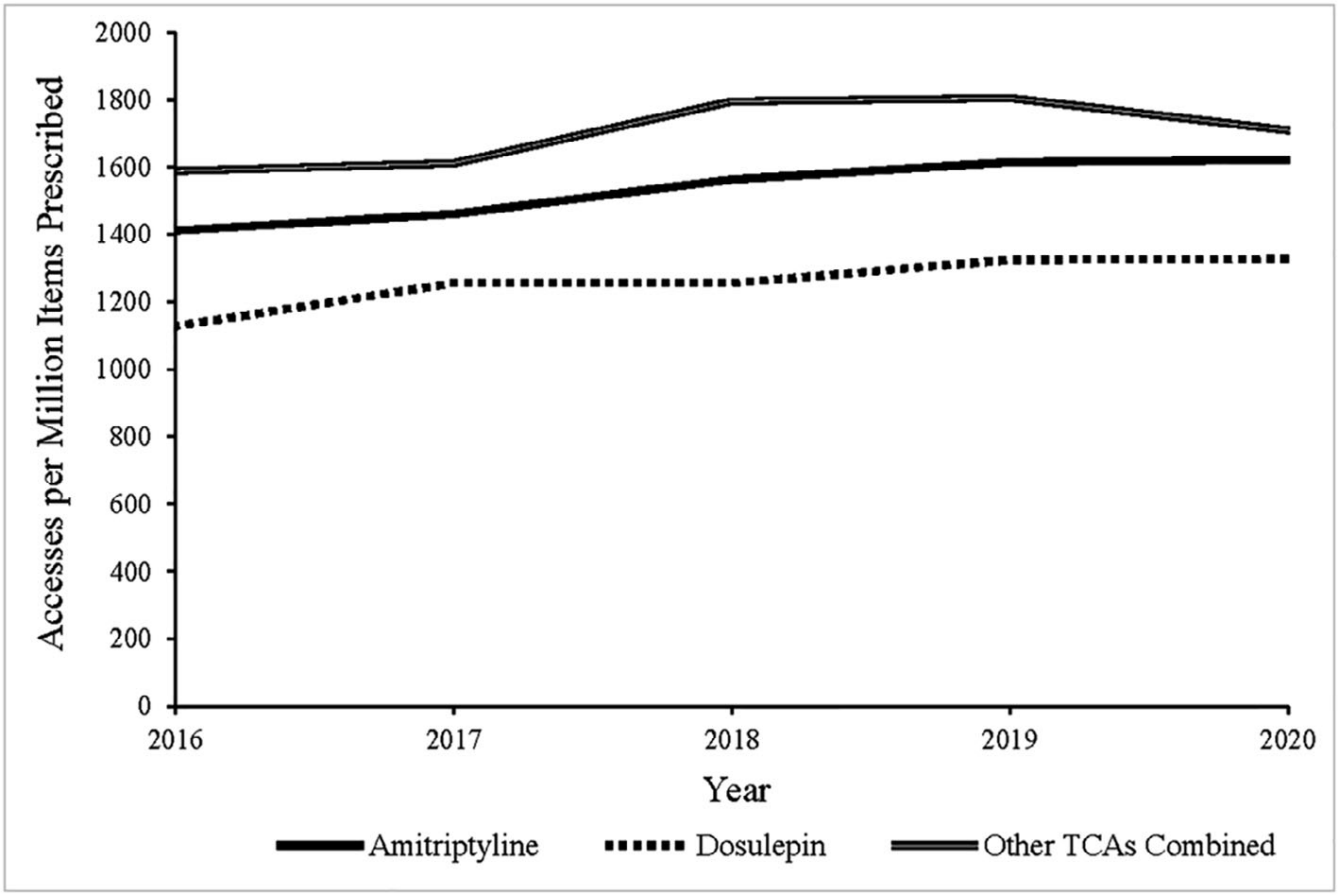
Annual Toxbase™ tricyclic antidepressant (TCA) resource web and app accesses per million TCA items dispensed in England and Wales 2016–2020.

Accesses of Toxbase™ TCA guidance rose over the data period (Figure 5). Although amitriptyline had the highest number of accesses for all years of the data period, scaling the data to the yearly items dispensed revealed that there were more accesses per million items dispensed with amitriptyline than seen with dosulepin. Both dosulepin and amitriptyline had fewer accesses per million items dispensed than the mean of the remaining six TCA drugs, however.

### 3.5 HES and PEDW data

The analysis of finished consultant episodes (FCEs) across the study period revealed a decrease in the number of FCEs in 2020–21 compared to 2015–16 in both England and Wales. In England, tricyclic and tetracyclic antidepressant poisoning‐related FCEs (TTFCEs) decreased by 15.6%, with 4126 cases in 2015–16, declining to 3483 in 2020–21. TTFCEs in Wales fell by 9.6% over the same period, with 156 in 2015–16 and 141 in 2020–21.

**FIGURE 6:**
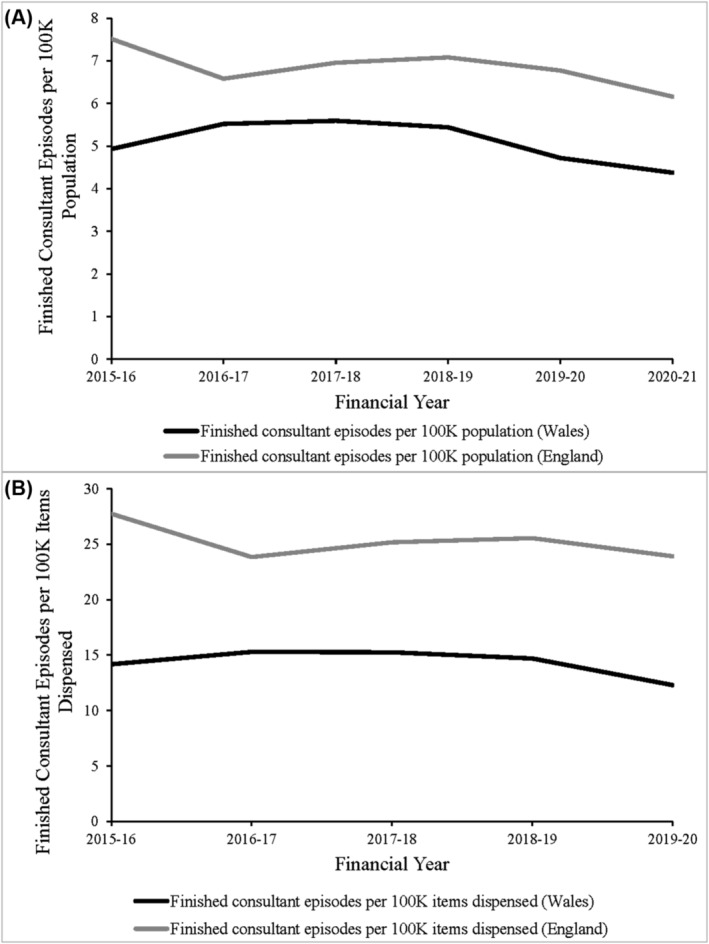
Secondary care tricyclic antidepressant (TCA) data. Finished consultant episodes in England and Wales where the primary diagnosis was recorded as T43.0: Poisoning: Tricyclic and tetracyclic antidepressants, scaled to (A) the population of each nation and (B) the total number of tricyclic antidepressant items dispensed in primary care in that financial year.

Scaling TTFCEs to the respective populations of England and Wales revealed a disparity in the number of TTFCEs per 100 000 population in each nation (Figure 6). England recorded more TTFCEs per 100 000 population across all years studied, with mean annual totals 134.3% higher than those observed in Wales. The TTFCEs per 100 000 population in England were 7.51 in 2015–16 and decreased by 18.0% to 6.16 in 2020–21. In Wales, they fell by 11.2%, from 4.93 TTFCEs per 100 000 population in 2015–16 to 4.38 in 2020–21. Scaling TTFCEs to the number of items dispensed revealed a similarly decreasing trend in both England and Wales over the data period (Figure 6). Notably, the disparity between the two nations was greater when scaled this way, with England recording a mean annual TTFCE level 176.0% that of Wales over the 5‐year period.

## 4 DISCUSSION

Despite an increase in the prevalence of depression,^44^, ^45^ a fall in dispensing in six of the eight TCAs was seen in England and Wales across the data period, potentially an ongoing effect of the 2009 NICE guideline limiting TCA use in depression,^3^ though the absence of data between 2009 and 2015 in this study means this link cannot be made with certainty. Dosulepin showed the greatest proportional decrease in dispensing amongst all TCAs, with the 2007 MHRA Drug Safety Update^18^ potentially driving prescriber behaviour in both nations. In Wales, the introduction of a specific prescribing indicator in 2011 was also associated with reduced dosulepin prescribing prior to 2016.^20^

Management of neuropathic and chronic pain is therapeutically challenging. Neuropathic pain has been estimated to affect 6.9–10% of the population^46^ and prevalence increases with age.^47^, ^48^ The prevalence of chronic pain in the UK has previously been estimated at 43%, and is expected to increase further in line with an ageing population.^49^ Our study revealed increased dispensing of amitriptyline and nortriptyline, the two TCAs used to treat neuropathic and chronic pain in England and Wales.^50^ Furthermore, amitriptyline's increased dispensing was almost exclusively in the lower‐strength formulations indicated in treating neuropathic and chronic pain,^10^ providing further evidence that this indication is driving TCA demand. With the prevalence of neuropathic pain likely to increase given the UK's ageing population, it remains vital that prescribing of TCAs and their effects are closely monitored to ensure that any potential harm is reduced. Concerns over the increased prescribing of gabapentin and pregabalin have led to their reclassification as a “controlled drug” in 2019 and a recent study has shown prescribing has plateaued since this change.^50^ It is important that if gabapentinoid prescribing becomes more restricted, that TCAs do not fill the “therapeutic gap”. Closer monitoring of trends in prescribing in TCAs, greater access to pain specialists and utilization of non‐pharmacological methods may ameliorate future rises in TCA prescribing and poisonings.

Telephone enquiries to the NPIS regarding TCAs fell over the data period, whilst the number of Toxbase™ web and app accesses for TCAs rose. Increased prescribing of amitriptyline and nortriptyline in lower‐strength formulations may increase poisonings via increased availability but produce fewer poisonings that require poisons specialist input via the NPIS. It is possible that less severe cases are manageable with available Toxbase™ guidance alone and a rising number of non‐severe poisonings are being handled using these resources. Thus, despite an increase in the use of amitriptyline and nortriptyline, the reduction in formulation strength may have ameliorated overdose severity. This was also supported by the decreased number of FCEs related to tricyclic and tetracyclic antidepressants seen in the PEDW and HES datasets for Wales and England respectively. Fewer FCEs suggests that either fewer poisonings are happening, or where they are happening, they do not cross the threshold for clinical treatment. The increase in accesses to TCA Toxbase™ guidance suggests more frequent utilization of Toxbase™ by clinicians for guidance on the management of this smaller pool of admitted cases.

The UKPID dataset provided insightful clinical information and allowed valuable analysis into the circumstances, patient demographics and severity of TCA poisonings. Intentional poisonings were the most common circumstance, emphasizing the role of TCAs in deliberate self‐harm. The majority of TCA poisoning patients being female is in keeping with UK self‐poisoning epidemiology.^51^, ^52^ A higher prevalence of both neuropathic pain and depression has been reported in UK women, providing further evidence that these conditions may drive TCA access and use.^49^, ^53^

When considering median poisoning doses of amitriptyline and their relation to MaxPSS score, those who were symptomatic (MaxPSS ≥ 1) had a dose close to, but above the referral level of 3 mg kg^‐1^,^54^ supporting the current referral threshold in Toxbase™. Limited comparable data for the other TCAs meant that equivalent analysis was not possible.

The number of deaths recorded in the UKPID dataset was lower than that reported by the ONS. Those found deceased following a TCA poisoning will be reported in the ONS dataset but not the UKPID dataset as no clinical input is required. An encouraging trend of declining deaths associated with TCAs in both England and Wales was seen over the study period. ONS mortality data for specific TCAs beyond amitriptyline and dosulepin was not available. Given the relatively low dispensing rates of other TCAs, it is unlikely that their inclusion would provide additional meaningful insights, as was true for TCA UKPID enquiries. However, the ONS database remains a useful resource for monitoring trends in causes of death from TCAs more broadly.

### 4.1 Limitations and future work

This study provides valuable insights into TCA prescribing and poisoning trends in England and Wales, but several limitations are noted. The exclusion of Scotland and Northern Ireland from the analysis limits the generalizability of the findings to the UK as a whole. Future research could include data from these nations to allow a more comprehensive understanding and to ensure UK‐wide guidelines are fit for purpose.

The TCA prescribing data utilized related to “items” rather than the quantity prescribed. While this approach may introduce data skewing if small quantities are prescribed frequently, it was mitigated by the fact that TCAs are commonly used long‐term for chronic conditions. The use of dispensed “items” as a proxy for TCA usage also does not account for whether medications were taken as prescribed. Future studies could link prescription data to adherence patterns and outcome measures.

The NPIS relies on healthcare professionals to make enquiries in order for cases to be recorded in the UKPID, and therefore not all poisonings will be captured. Clinically straightforward cases are less likely to require specialist input and so will likely be disproportionately underreported. The limited follow‐up of NPIS poisoning cases (5.4%) restricts the understanding of long‐term outcomes. Future research should prioritize more comprehensive case follow‐up to allow a more holistic clinical analysis of poisonings. Limited demographic data amongst UKPID enquiry entries also mean that links between socioeconomic factors and TCA poisoning risk could not be determined, but this represents a useful avenue for future study. It was also not possible to conduct robust comparisons of dose ingestion between TCAs beyond amitriptyline due to the low number of poisoning enquiries received for these drugs. Additionally, while the increase in Toxbase™ accesses may indicate that more clinicians are seeking guidance due to the rising use of TCAs, it is also used for educational purposes and so not all accesses will be the result of poisonings.

ONS mortality data has limitations including reporting delays, inaccurate cause of death information, and potential coding inconsistencies, all of which can affect the observed trends.^51^ Statistics are based on the year of death registration, but because of death registration delays, around half of these deaths will have occurred in previous years. Specific causative agents are not always recorded within the ONS poisonings dataset, and so deaths relating to amitriptyline and dosulepin may have been higher than reported. More than half of all drug‐poisoning deaths involve more than one drug, and it is not always possible in those cases to tell which substance was primarily responsible for the death.^55^ The smaller population of Wales, and therefore lower absolute number of TCA deaths, introduced greater volatility into the trend of population‐scaled deaths than amongst the equivalent English data. The magnitude of the difference in the trends of TCA deaths (decreases of 73.9% and 2.9% for Wales and England respectively) suggests that a real difference exists, however. The incomplete coding of causes of poisoning in hospital data can also lead to underreporting of poisonings. While the T430 ICD‐10 code provides a standardized approach for identifying TCA poisonings in HES/PEDW, there is potential for misclassification or under‐detection due to variability in clinical documentation and coding practices.

Lastly, we note that the early stages of the COVID‐19 pandemic and national lockdowns did not produce noticeable deviations from the existing trends in the datasets we analysed. The long‐term effects of the pandemic on TCA use and poisoning patterns remain uncertain and further research is needed to explore these impacts as further data become available.

## 5 CONCLUSION

This study found encouraging evidence that the implementation of tricyclic antidepressant (TCA) prescribing guidelines and utilization of lower‐strength formulations were associated with a fall in both the number of clinically severe poisonings and deaths despite the overall increase in dispensing of TCAs. A reduction in dispensed formulation strength should therefore be considered as an option in reducing harm from other medications. Regarding clinical management, current referral doses in Toxbase™ for amitriptyline appear appropriate.

## AUTHOR CONTRIBUTIONS

Laurence Gray, Michael Beddard and Matthew O. Ivory designed the study. Michael Beddard, Asiyah Begum and Noraini B. Azhar analysed the ONS datasets. Laurence Gray and Michael Beddard undertook the processing and analysis of anonymized UKPID enquiry data. Matthew O. Ivory and Paul Deslandes analysed primary care prescribing data. Matthew O. Ivory, Laurence Gray, Michael Beddard and Stephen Jones analysed secondary care data. Stephen Jones performed trend analysis and statistical tests. All authors contributed to the interpretation of results, writing and reviewing the manuscript and gave approval for publication.

## CONFLICT OF INTEREST STATEMENT

There are no competing interests to declare.

## Supporting information


**Figure S1** Visualization of the datasets analysed within the study.

**Figure S2** Trend analysis of population‐scaled monthly English and Welsh amitriptyline primary care items dispensed classified by tablet strength.

**Figure S3** Trend analysis of population‐scaled monthly English and Welsh primary care items dispensed for the seven non‐amitriptyline UK‐licensed tricyclic antidepressants.

**Table S1** Model coefficients from linear regression analyses for each tricyclic antidepressant to illustrate national differences. The dependent variable is number of items prescribed per 100 000 population and the independent variable is time by month over the study period. Positive values for the change per month over the time period indicate increases in items prescribed for that drug, negative changes indicate decreases. Values in brackets are 95% confidence intervals. *P*‐values of less than 0.05 indicate the estimated monthly change is statistically significantly different from zero.

## Data Availability

Data sources are provided in the text.
