# Supplementary figures and images for: Trends in tricyclic antidepressant prescribing and poisoning in England and Wales 2016–2020
Source: Br J Clin Pharmacol. 2025 Jan 29;91(6):1727–38. doi: 10.1111/bcp.16400 (PMC12122128; doi:10.1111/bcp.16400)

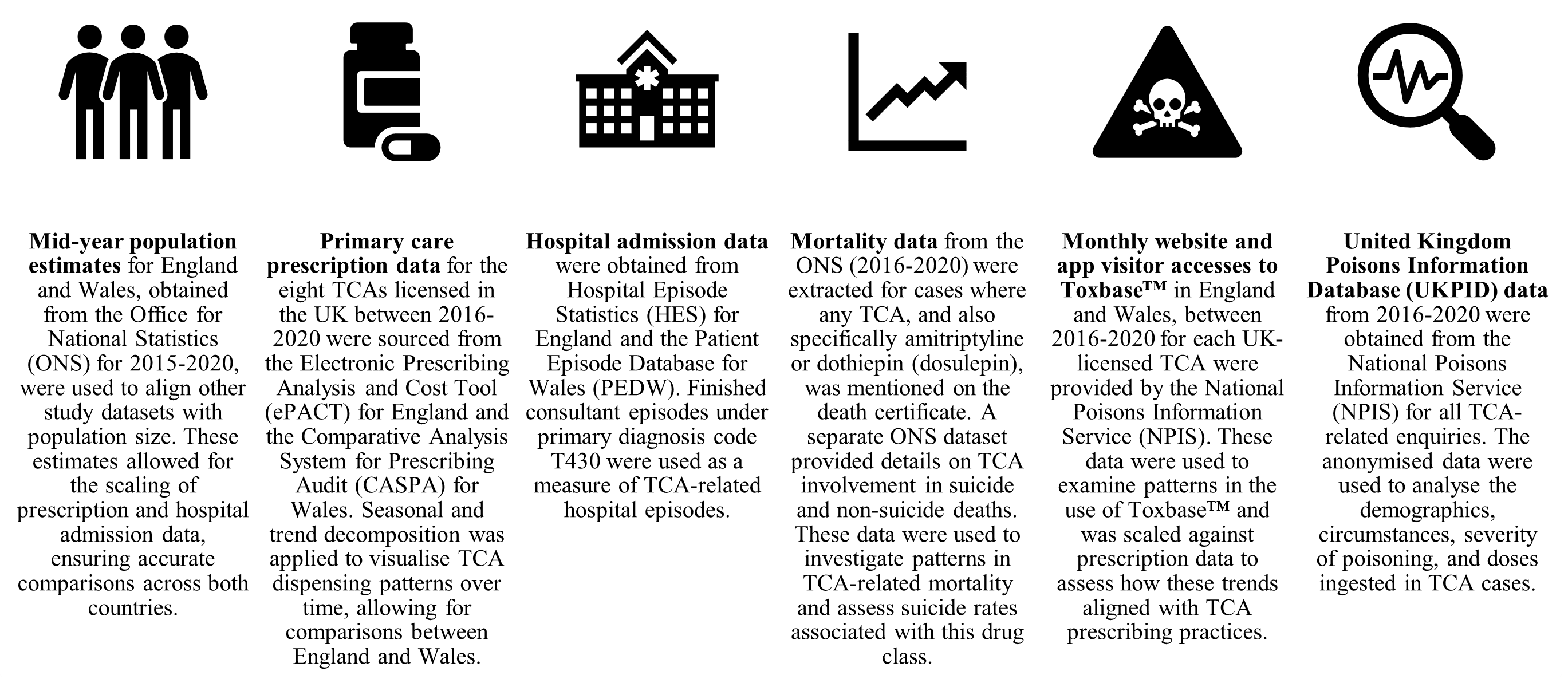

Supplement: Supplementary file 1 — Figure S1 Visualization of the datasets analysed within the study. [file BCP-91-1727-s001.tif]

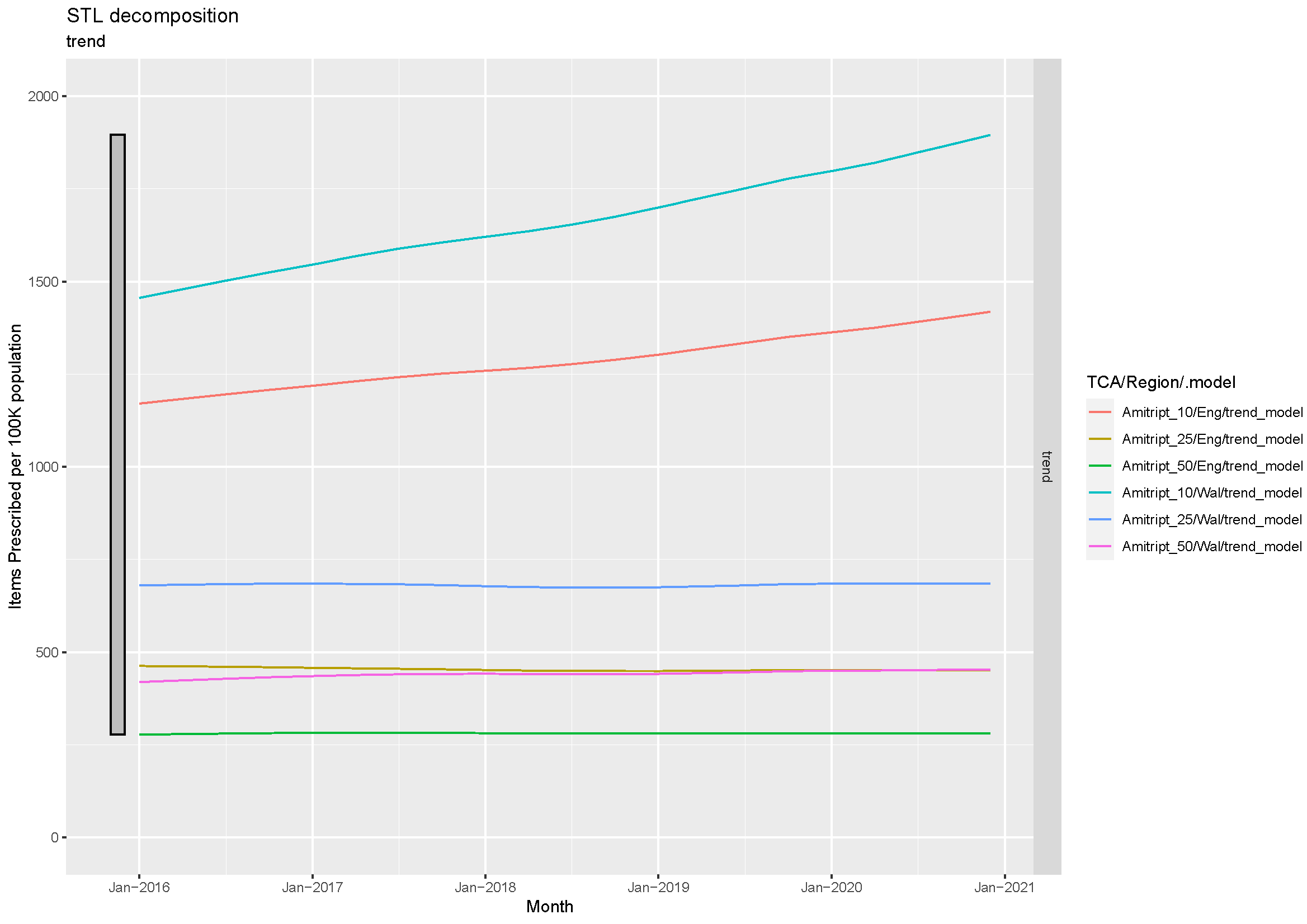

Supplement: Supplementary file 2 — Figure S2 Trend analysis of population‐scaled monthly English and Welsh amitriptyline primary care items dispensed classified by tablet strength. [file BCP-91-1727-s004.tif]

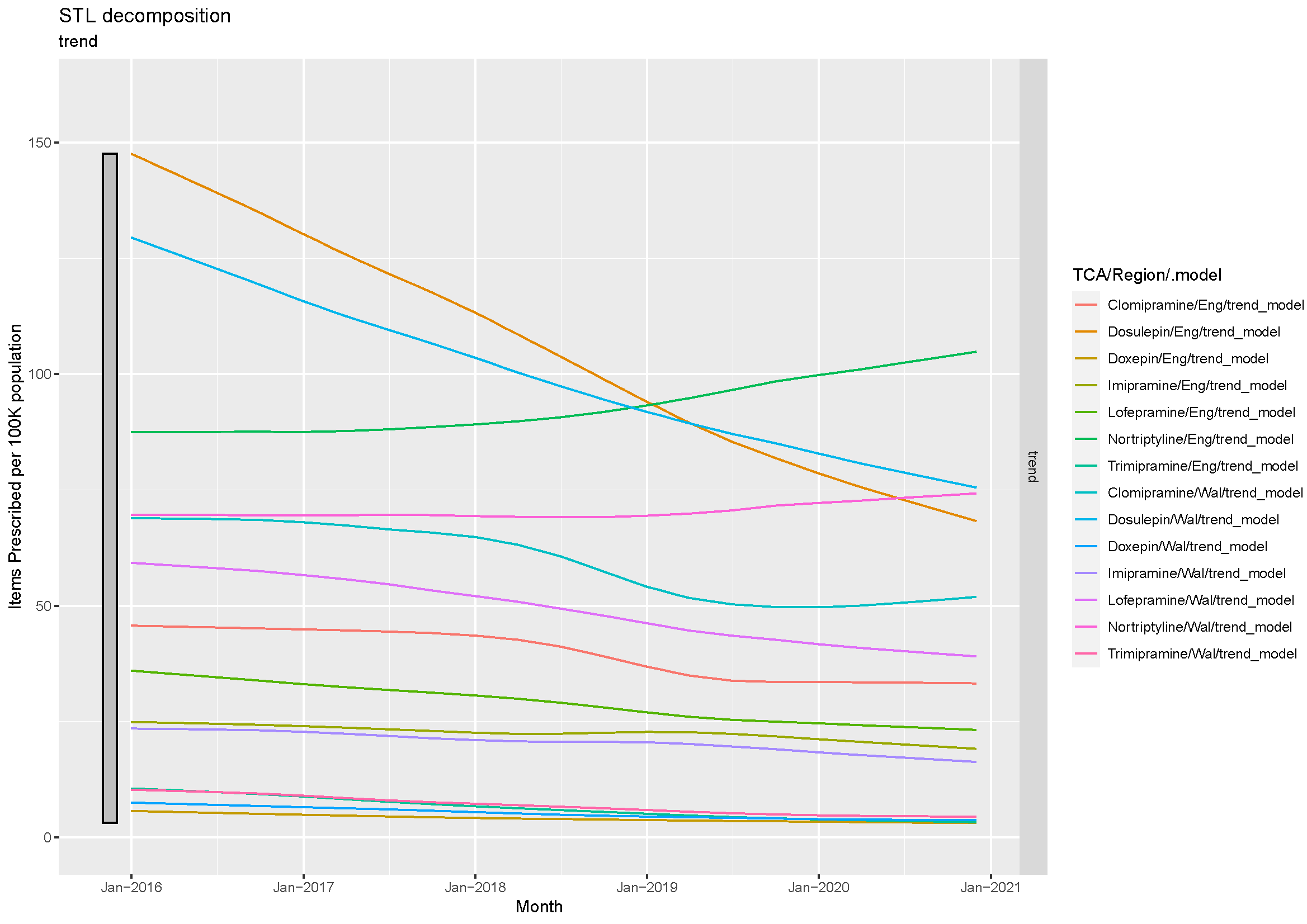

Supplement: Supplementary file 3 — Figure S3 Trend analysis of population‐scaled monthly English and Welsh primary care items dispensed for the seven non‐amitriptyline UK‐licensed tricyclic antidepressants. [file BCP-91-1727-s003.tif]
