# Supplementary material for: Trends in tricyclic antidepressant prescribing and poisoning in England and Wales 2016–2020
Source: Br J Clin Pharmacol. 2025 Jan 29;91(6):1727–38. doi: 10.1111/bcp.16400 (PMC12122128; doi:10.1111/bcp.16400)
Supplement: Supplementary file 4 — Table S1 Model coefficients from linear regression analyses for each tricyclic antidepressant to illustrate national differences. The dependent variable is number of items prescribed per 100 000 population and the independent variable is time by month over the study period. Positive values for the change per month over the time period indicate increases in items prescribed for that drug, negative changes indicate decreases. Values in brackets are 95% confidence intervals. P‐values of less than 0.05 indicate the estimated monthly change is statistically significantly different from zero. [file BCP-91-1727-s002.pptx]

## Slide 1
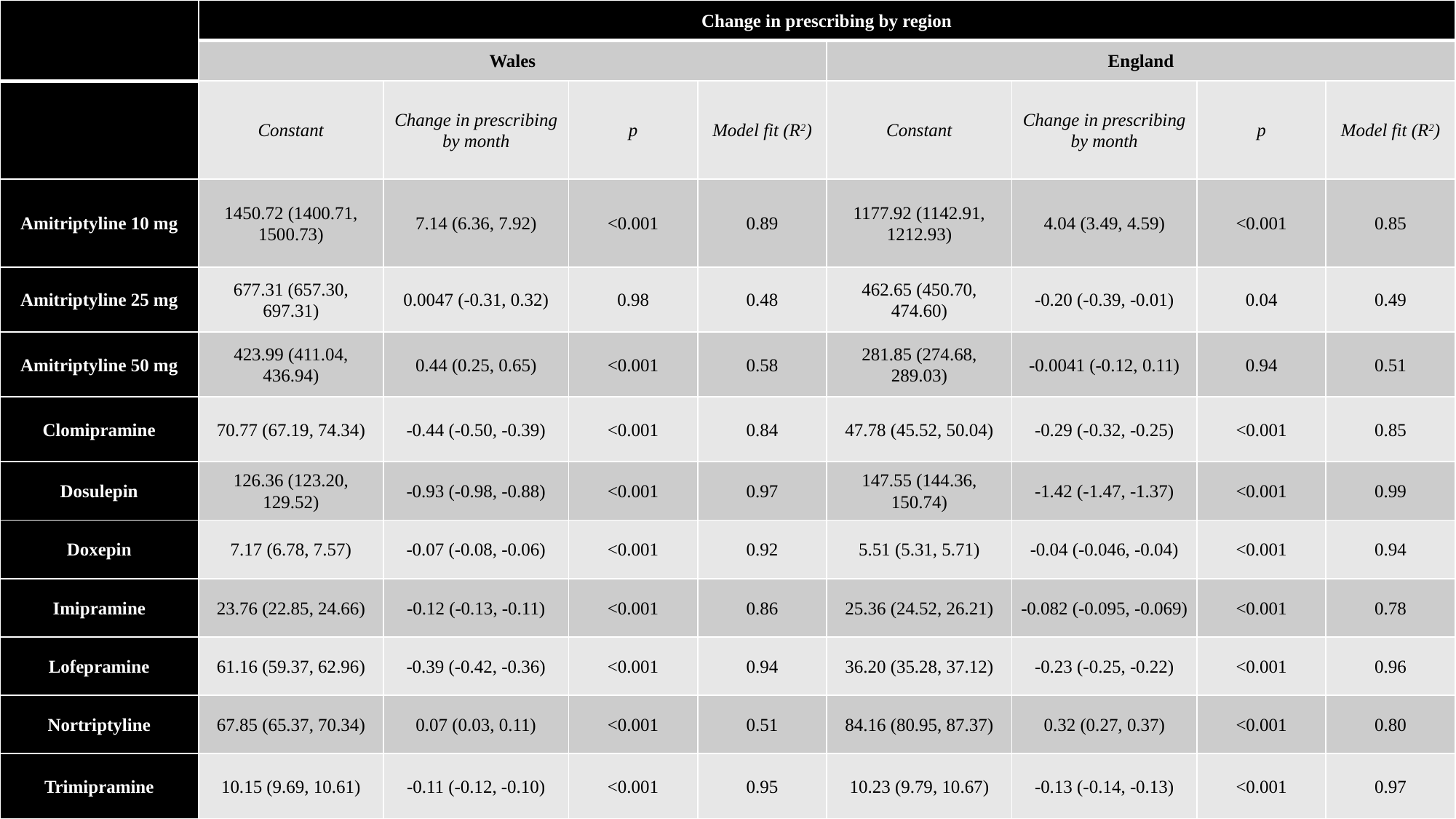

| | Change in prescribing by region | | | | | | | |
| --- | --- | --- | --- | --- | --- | --- | --- | --- |
| | Wales | | | | England | | | |
| | Constant | Change in prescribing by month | p | Model fit (R2) | Constant | Change in prescribing by month | p | Model fit (R2) |
| Amitriptyline 10 mg | 1450.72 (1400.71, 1500.73) | 7.14 (6.36, 7.92) | <0.001 | 0.89 | 1177.92 (1142.91, 1212.93) | 4.04 (3.49, 4.59) | <0.001 | 0.85 |
| Amitriptyline 25 mg | 677.31 (657.30, 697.31) | 0.0047 (-0.31, 0.32) | 0.98 | 0.48 | 462.65 (450.70, 474.60) | -0.20 (-0.39, -0.01) | 0.04 | 0.49 |
| Amitriptyline 50 mg | 423.99 (411.04, 436.94) | 0.44 (0.25, 0.65) | <0.001 | 0.58 | 281.85 (274.68, 289.03) | -0.0041 (-0.12, 0.11) | 0.94 | 0.51 |
| Clomipramine | 70.77 (67.19, 74.34) | -0.44 (-0.50, -0.39) | <0.001 | 0.84 | 47.78 (45.52, 50.04) | -0.29 (-0.32, -0.25) | <0.001 | 0.85 |
| Dosulepin | 126.36 (123.20, 129.52) | -0.93 (-0.98, -0.88) | <0.001 | 0.97 | 147.55 (144.36, 150.74) | -1.42 (-1.47, -1.37) | <0.001 | 0.99 |
| Doxepin | 7.17 (6.78, 7.57) | -0.07 (-0.08, -0.06) | <0.001 | 0.92 | 5.51 (5.31, 5.71) | -0.04 (-0.046, -0.04) | <0.001 | 0.94 |
| Imipramine | 23.76 (22.85, 24.66) | -0.12 (-0.13, -0.11) | <0.001 | 0.86 | 25.36 (24.52, 26.21) | -0.082 (-0.095, -0.069) | <0.001 | 0.78 |
| Lofepramine | 61.16 (59.37, 62.96) | -0.39 (-0.42, -0.36) | <0.001 | 0.94 | 36.20 (35.28, 37.12) | -0.23 (-0.25, -0.22) | <0.001 | 0.96 |
| Nortriptyline | 67.85 (65.37, 70.34) | 0.07 (0.03, 0.11) | <0.001 | 0.51 | 84.16 (80.95, 87.37) | 0.32 (0.27, 0.37) | <0.001 | 0.80 |
| Trimipramine | 10.15 (9.69, 10.61) | -0.11 (-0.12, -0.10) | <0.001 | 0.95 | 10.23 (9.79, 10.67) | -0.13 (-0.14, -0.13) | <0.001 | 0.97 |
